# Carbon sinks and carbon emissions balance of land use transition in Xinjiang, China: differences and compensation

**DOI:** 10.1038/s41598-022-27095-w

**Published:** 2022-12-27

**Authors:** Kui Luo, Hongwei Wang, Chen Ma, Changrui Wu, Xudong Zheng, Ling Xie

**Affiliations:** 1 College of Geography and Remote Sensing Sciences, Xinjiang University, Urumqi, 830017 China; 2 Xinjiang Key Laboratory of Oasis Ecology, Xinjiang University, Urumqi, 830017 China; 3 School of Geography and Ocean Science, Nanjing University, Nanjing, 210023 China

**Keywords:** Ecosystem services, Environmental economics, Sustainability

## Abstract

With the continuous enhancement of human activities, the contradiction between regional development and ecological protection is prominent in the ecologically fragile arid areas. It is of great significance for regional sustainable development to understand the ecological supply and demand problems caused by transformation of land using and formulate ecological compensation scheme scientifically. This study takes Xinjiang in China as the research area. It explores the land use transition characteristics and the changes in carbon supply and demand of Xinjiang using methods such as GIS spatial analysis and modified comparative ecological radiation forcing. Finally, the ecological compensation scheme is studied based on the theory of ecological radiation. The research shows that (I) in the study chronology, most of the areas produced only one change in land use. Land use is gradually developing towards the direction of ecological protection. After 2000, grassland recovered well, and 14,298 km^2^ of other ecological land was transformed into grassland. (II) The change in the carbon sink of the Xinjiang ecosystem first decreased and then increased, and the ecological deficit area started to appear after 2010. The growth of grassland and cropland areas is essential to enhance the carbon sink capacity of arid zones. (III) The amount of ecological compensation in Xinjiang is 31.47 * 10^8^ yuan, and the proportion of the amount received by ecological compensation areas is related to the distance between the supply and demand areas, the amount of carbon sequestration, and the area of the region. This study provides a reference for achieving the healthy development of sustainable land use ecosystems in arid zones.

## Introduction

The International Geosphere-Biosphere Programme (IGBP) and the International Human Dimension Programme on Global Environmental Change (IHDP) proposed a core research program on land use and cover change (LUCC) in 1995^1,2^. LUCC is treated as a coupled human-land system that addresses theories, concepts, and models related to environmental and social issues^3^. The report issued by the Intergovernmental Panel on Climate Change (IPCC) in 2006 stated that LUCC caused carbon to be released into the atmosphere at a rate of 1.5 Pg per year^4^, indicating that the role of LUCC in carbon sinks in terrestrial ecosystems has changed dramatically. Carbon sinks are long-term carbon reservoirs, including soil, plant and other forms; carbon sinks mitigate climate change and offset anthropogenic carbon emissions^5^. Carbon emissions are entities that emit carbon dioxide into the atmosphere, resulting in an increase in the concentration of carbon dioxide in the atmosphere^6^. In China, uncontrolled human interference in nature during recent decades, especially those before 2000, has led to the extensive degradation of land with high carbon sequestration capacity, thus affecting the carbon sink capacity of ecosystems^7^. At the same time, China has surpassed the United States as the world's largest emitter of carbon dioxide, as rapid economic development has led to increased fossil energy consumption and per capita carbon emissions^8,9^. Based on these circumstances, the Chinese government has set the development goals of peaking carbon emissions by 2030 and achieving a carbon balance by 2060^10^, leaving time and options for further improvement of ecological governance efficiency. Clarifying the supply–demand-compensation relationship between LUCC and carbon sequestration in terrestrial ecosystems is important to achieve effective management of land resources and the goal of synergistic development of regional ecology and economy^11,12^.

Ecosystem services refer to the benefits of ecological processes and their effectiveness for human well-being^13^. Carbon sinks are an important ecological regulation service closely related to human activities^14^. Land is an important carrier of natural carbon sinks and anthropogenic carbon emissions, and land cover change is influenced by both climate and human activities^15^. Land use transition is regarded as a new aspect of LUCC research^16^, including the process of temporal change in the explicit and implicit forms of land use^17^. Land use transition is one of the important causes of changes in ecological environment effects, affecting the ability of the ecological environment to provide more ecosystem services for sustainable development^18,19^. In addition, spatial changes in land use reflect changes in human socioeconomic activities, usually in the form of energy consumption and thus carbon emission growth^20^. In reality, the spatial heterogeneity in the distribution of ecosystem services and land resources and changes in human demand often leads to a mismatch between supply and demand of ecosystem services in local areas, and this imbalance causes a deficit in ecosystem services^7^. Externalities of ecosystem services lead to environmental inequities, as ecosystem services generated in a given region have extra-regional utility, but the scope and scale of services provided directly to beneficiaries vary greatly from region to region^21^. Payments for ecosystem services can transform uncompensated externalities into economic incentives and are important for protecting ecosystems and coordinating regional development^22,23^. At present, approximately 56 countries around the world have established and implemented ecological compensation policies, and ecological compensation is mostly government-led^24^. China's ecological compensation system began in the 1990s with the return of farmland to forests, and a new round of ecological compensation programs was implemented in 2014^6,25,26^.

Current research focuses on studying land use transition through tools such as RS and GIS^17,27^ and measuring the impact of land use change on regional ecology using indicators such as the value of ecosystem services^10,28^, assessment of the actual function of ecosystem services^29,30^, and remote sensing index of the ecological environment^31,32^. Research methods are mainly divided into field sampling and analysis, modeling using ecological models, and inversion of remote sensing images^29,33–35^. In recent years, worldwide, governments have reached consensus on the need to reduce carbon emissions and enhance carbon sequestration to combat global warming, and research on land use transition and carbon sequestration has received extensive attention from scholars^34,36–38^. Most current studies use IPCC carbon inventories and factors or per capita carbon emissions to account for land carbon emissions^25,39^. Studies have shown that environmentally friendly anthropogenic activities such as afforestation can enhance ecological carbon sink functions through ecological conservation^40^. Converting unused land to cropland is also considered an effective strategy for mitigating carbon emissions, and cropland for growing crops is an important carbon sink for drylands^41^. However, with concentrated population and economic growth, ecosystem demands are gradually being incorporated into the research system, and regional ecological development potential is often measured by the difference in ecological supply and demand^6,26^. As one of the important means to alleviate the contradiction between ecological supply and demand, ecological compensation needs to be based on factors measured by actual prices. For example, ecological compensation can be realized in reality by accounting for water supply and demand and carbon supply and demand via ecological radiation, corridor identification and other methods^11,25,42^.

**Figure 1:**
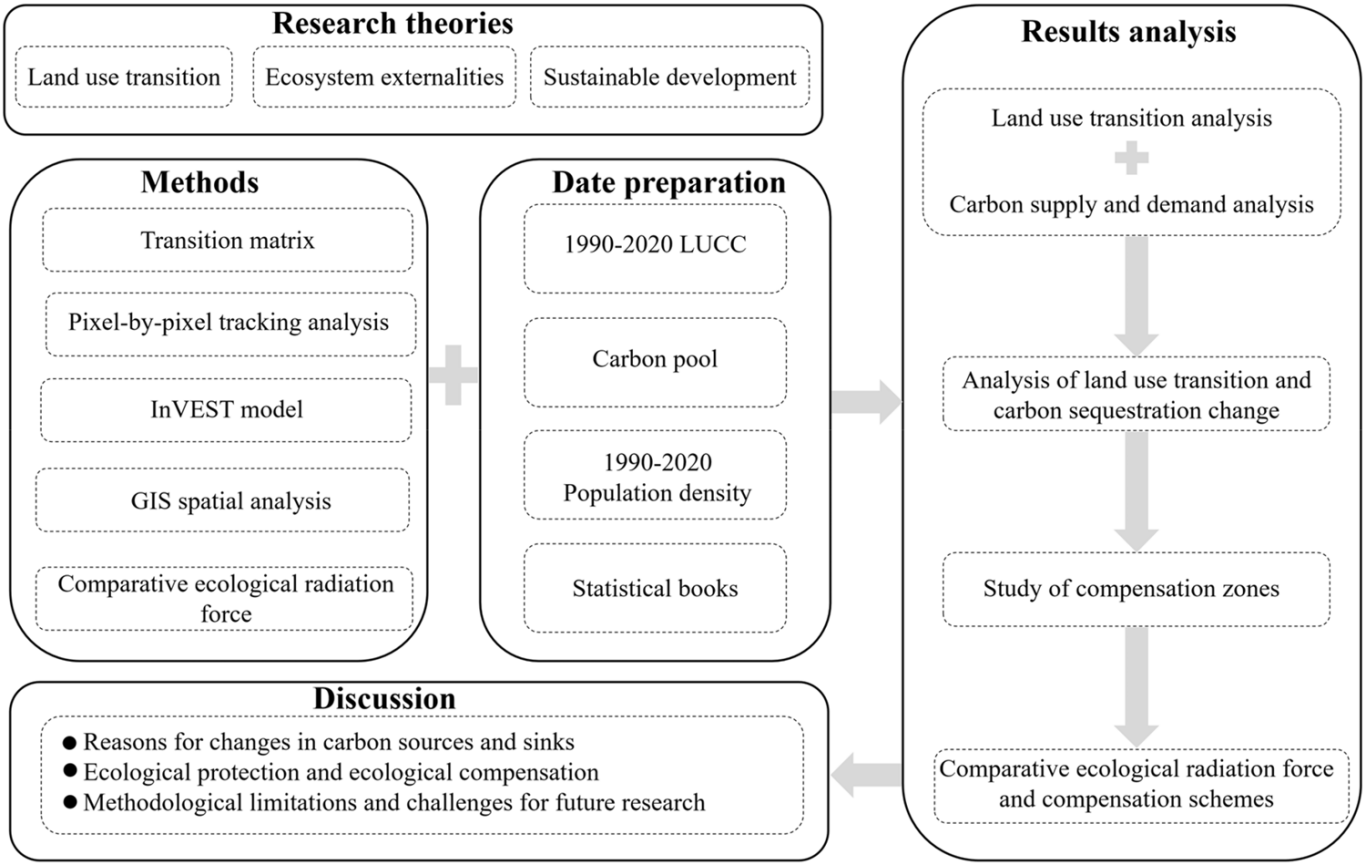
Research framework of this study. Software: PowerPoint 2021. URL: https://www.microsoft.com/zh-cn/.

Arid areas account for approximately 35% of the world's land and are home to nearly one-third of the world's population, but the ecological environment in arid areas is very fragile^43^. Human activities have put enormous pressure on the ecological environment in arid areas, though the ecosystems in these areas provide environmental resources for maintaining well-being. Xinjiang is a typical arid and ecologically fragile area in China, but it is rich in energy such as oil and gas. In recent years, excessive reclamation of oases and the accelerated development of urbanization, have caused serious damage to the ecological environment, eventually leading to a decline in the value of ecosystem services^44,45^. Most studies regarding the impact of land use transformation on ecosystem services in Xinjiang focus on the accounting of ecosystem service value^46^. In addition, some studies focus on simulating changes in ecosystem services by predicting future land change in Xinjiang^47,48^. At present, research on ecosystem services in Xinjiang mainly focuses on the evaluation of supply capacity and pays less attention to the relationship between supply and demand. Research on ecological compensation is at the initial stage. The county is the main administrative unit in China and offers the optimal scale for environmental planning and management, and the basic unit for ecosystem service evaluation^49^. Based on the above discussion, the research purposes of this paper are as follows (Fig. 1): (I) to explore the process of land use change in Xinjiang from 1980 to 2020, summarize the characteristics of land use transition, and summarize the changes in key ecological protection land types such as forestland and grassland and land use for human activities; (II) to evaluate the evolution of ecosystem carbon supply and demand in Xinjiang counties from 1990 to 2020, analyze areas with ecological deficits and surpluses, and conduct joint analysis with land use transitions; and (III) to exploring the ecological compensation scheme under the difference of carbon sinks and carbon emissions in Xinjiang. This study is beneficial to reveal the changing patterns of carbon sinks and carbon emissions differences in Xinjiang in the context of land use transformation. The modified comparative ecological radiation model is used to spatially simulate the extra-territorial effects of ecosystem services in Xinjiang and propose an ecological compensation scheme. The research results can provide some scientific basis for the construction of sustainable management of land resources and ecological compensation schemes in Xinjiang, which is conducive to remedying the environmental equity problems brought about by economic development and provides some reference suggestions for the harmony between humans and nature in Xinjiang.

## Materials and methods

### Study area

**Figure 2:**
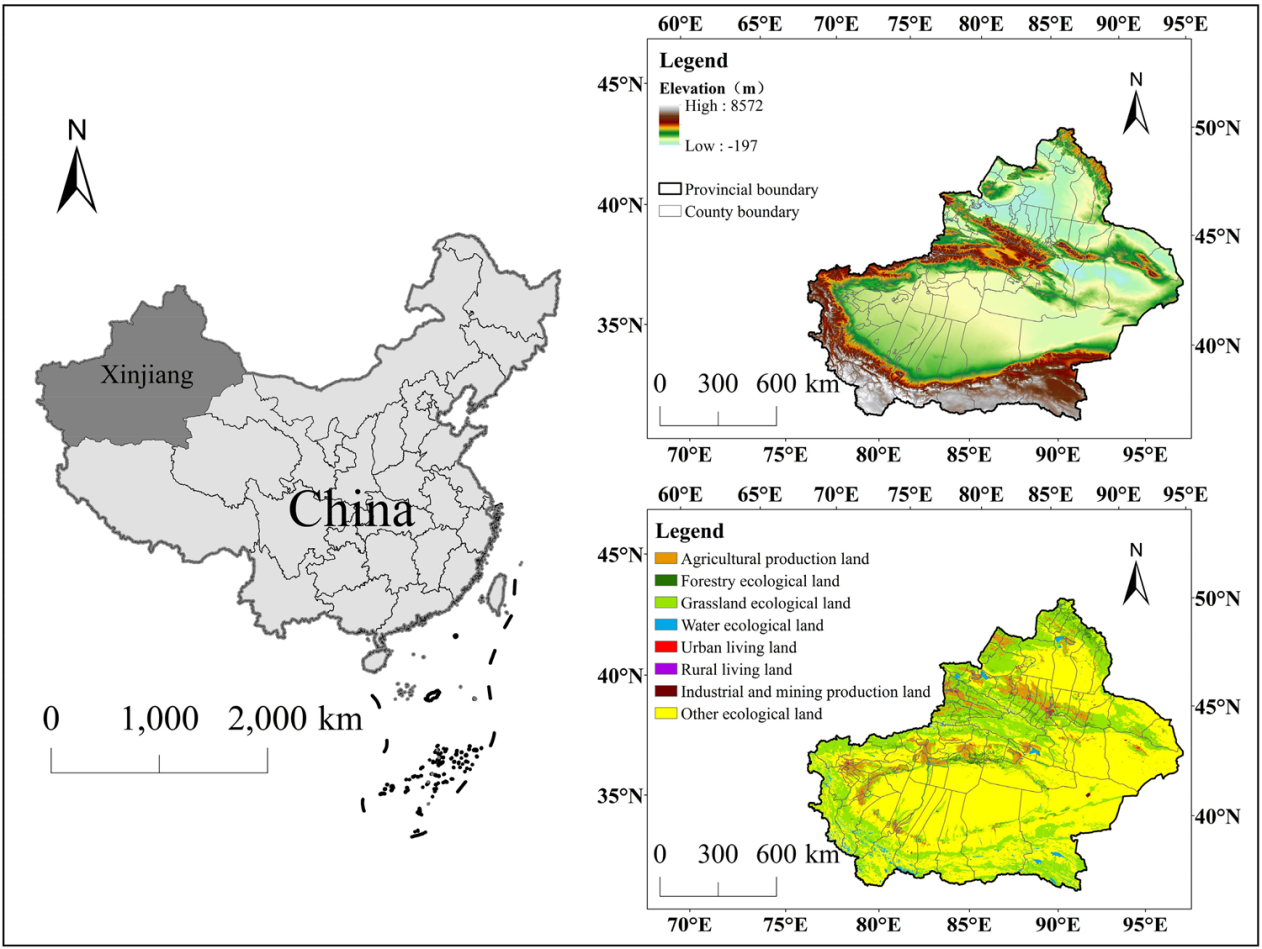
Location of the study area. Map generated with ArcGIS 10.8 (ESRI). URL: https://support.esri.com/en/Products/Desktop/arcgis-desktop/arcmap.

Xinjiang is located in the arid zone (34.25°N–49.17°N, 73.33°E–96.42°E) in the interior of northwest China (Fig. 2), and belongs to the central part of the Asia-Europe continent, covering approximately one-sixth of the land area of China^50^. Its main topographic feature is the alternating distribution of mountain basins. From north to south are the Altai Mountains, the Junggar Basin, the Tianshan Mountains, the Tarim Basin, and the Kunlun Mountains. There are deserts, Gobi, grasslands, forests, and other natural landscapes. Xinjiang has a temperate continental climate with strong evaporation, less precipitation in most areas, and an uneven spatial distribution of surface runoff. Water resources are one of the important factors restricting its development^45^. Xinjiang is an important ecological security area in the core economic belt of China's Silk Road, but its ecological environment is relatively fragile^51^. Since China's reform and opening up in 1978, with the deepening of policies such as the development of the western region, the aid plan for Xinjiang, and the construction of ecological civilization, Xinjiang's social economy has developed rapidly, and human activities have increased significantly. At the same time, the land use pattern in Xinjiang has undergone a major transformation, which has also had a greater impact on regional ecosystem services.

### Data sources and processing

The data used in this study and their sources are shown in Table 1. The land use data of the Resource and Environment Science and Date Center are based on Landsat TM/ETM+/OLI remote sensing images, which are generated by manual visual interpretation to achieve high accuracy with a raster spatial resolution of 30 m. Based on the natural attributes of land use and the dominant functions of land use, this study draws on relevant researches^45^, classifies land use according to ecological space (forestry ecological land, grass ecological land, water ecological land, other ecological land), production space (agricultural production land, industrial and mining production land) and living space (urban living land, rural living land). The Carbon Neutrality Research Database in the China Stock Market & Accounting Research Database (CSMR) accounts for carbon emissions through the IPCC inventory method. The Carbon Emission Accounts & Database (CEADs) study uses the particle swarm optimization-backpropagation (PSO-BP) algorithm to unify the scale of DMSP/OLS and NPP/VIIRS satellite images to estimate CO_2_ emissions^52^. The per capita carbon emission data of Xinjiang are obtained by dividing the carbon emissions by the population and using the population density map to map the carbon emissions in Arcgis10.8 for displaying and zoning statistics.

**Table 1:** Data sources.

Data type	Data source
Land use data	The Resource and Environment Science and Date Center (http://www.resdc.cn)
Elevation data	The Geospatial Data Cloud (https://www.gscloud.cn/)
Administrative map	The Resource and Environment Science and Date Center (http://www.resdc.cn)
Population density raster data	The Resource and Environment Science and Date Center (1990, 2000, 2010), World Pop(2020)
Carbon emissions data	The Carbon Emission Accounts & Database (CEADs) (https://www.ceads.net.cn/) (2000, 2010, 2020), The Carbon Neutrality Research Database in the China Stock Market & Accounting Research Database (CSMR) (https://www.gtarsc.com/) (1990)
National average price of carbon trading in 2020	China Carbon Emissions Trading Network (http://www.tanpaifang.com/)

### Methods

#### Transition matrix for land cover change detection

The transformation analysis of the land use function structure can be realized by the land use transition matrix model, which is derived from the quantitative description of the system state and state transition in the system analysis^53^. The land use transfer matrix is used to arrange the transfer area of each type of land use change in matrix form. The area comparison of any two phases of the land use transfer matrix can intuitively find the structural characteristics and land use function types of land use change^54^.

1 $${L}_{ij}=\left[\begin{array}{llll}{L}_{11} & \quad { L}_{12} & \quad \ldots & \quad  {L}_{1n}\\ {L}_{21} & \quad {L}_{22} & \quad \ldots & \quad { L}_{2n}\\ \ldots & \quad \ldots & \quad \ldots & \quad \ldots \\ {L}_{n1}& \quad {L}_{n2} & \quad \ldots & \quad  {L}_{nn}\end{array}\right]$$

where $L$ is the area of land type, $i \; and \; j$ are the land use types at the beginning and end of the study period, respectively, and $n$ is the number of land use types.

#### Pixel-by-pixel tracking analysis

**Figure 3:**
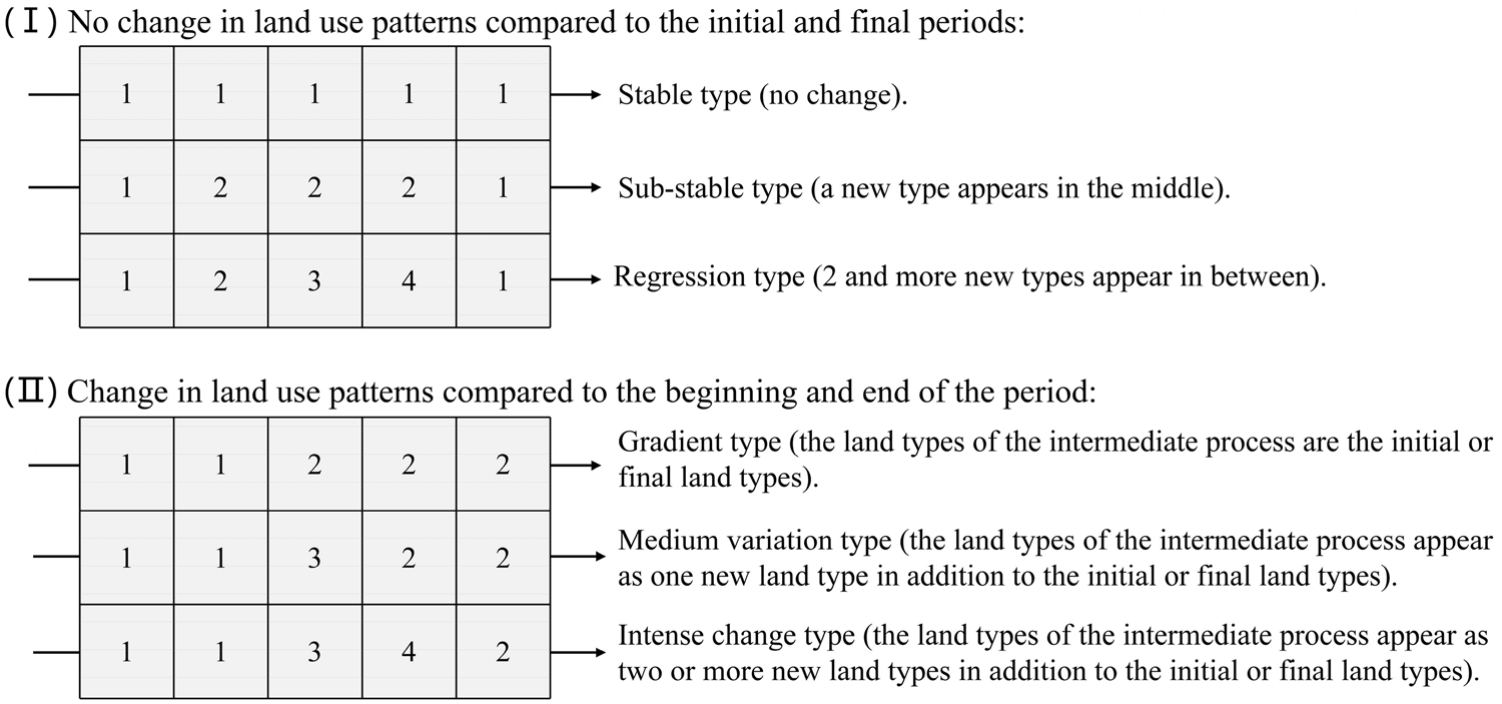
Types of land use change trajectory. Software: PowerPoint 2021. URL: https://www.microsoft.com/zh-cn/.

Pixel-by-pixel tracking analysis refers to analyzing the change trajectory of all land use types in a pixel within a certain period of time. This method can effectively avoid the inability to detect the intermediate change process in dual-phase change detection and reveal the spatial differentiation law of the land use transition process^55^. The pixel tracking analysis can be implemented in the spatial analysis function of Arcgis10.8. According to whether the land use change trajectory changes at the beginning and the end, and the severity of the process, the change trajectory is divided into 6 categories (Fig. 3)^56^.

#### Carbon supply and demand accounting

Various international scientific groups have advocated the Integrated Valuation of Ecosystem Services and Tradeoffs (InVEST) model as part of a new generation of ecosystem services assessment models, through which researchers can quantify and map carbon sequestration ecosystem services^34,57,58^. This study used the Carbon Sequestration (CS) module of the InVEST model to account for carbon sequestration and its changes in Xinjiang. The model calculates the total regional carbon stock through four carbon pools based on land use data. The carbon pool data used in this study were monitored from relevant experimental studies^6,59^; the sandy areas were listed separately because the recent studies have determined that the deserts in Xinjiang have some carbon sink capacity^60^. In this study, the data most suitable for the arid zone were selected, and the carbon density with multiple reference values were averaged to obtain the carbon density table for this study (Table 2). Based on the per capita carbon emissions and population density data, the carbon demand of each county in Xinjiang was obtained. According to the difference between supply and demand, the carbon ecological supply and demand value ($CSDR$) was obtained. If $CSDR>0$, the area is an ecological surplus area, and the supply is greater than the demand; otherwise, it is an ecological deficit area. The specific calculation formula is as follows.

**Table 2:** Carbon pool table (Mg hm^−2^ a^−1^).

Land use type	C_above	C_below	C_soil	C_dead
Agricultural production land	2.66	0.40	92.04	0.07
Forestry ecological land	44.60	11.10	106.10	1.90
Grassland ecological land	0.43	4.40	85.40	0.08
Water ecological land	0.00	0.00	0.00	0.00
Artificial surface	0.00	0.00	70.61	0.00
Desert	0.00	0.00	0.07	0.00
Other ecological land	0.00	0.00	0.00	0.00

Supply:

2 $${C}_{CS}=\left({C}_{a}+{C}_{b}+{C}_{s}+{C}_{d}\right)\times A$$

Demand:

3 $${C}_{CD}={C}_{p}\times {P}_{d}$$

4 $$CSDR={C}_{CS}-{C}_{CD}$$

where ${C}_{CS}$ is the sum of carbon sequestration by all land types, ${C}_{a}$, ${C}_{b}$, ${C}_{s}$, and ${C}_{d}$ are the carbon density in aboveground biomass, the carbon density in belowground biomass, the carbon density in soil, and the carbon density in dead organisms, respectively, and $A$ is the area of various land uses. ${C}_{CD}$ is the total amount of carbon emissions, that is, the demand for carbon sequestration ecosystem services, ${C}_{p}$ is the regional per capita carbon emissions, and ${P}_{d}$ is the regional population density. $CSDR$ is the regional carbon ecological supply and demand difference.

#### Contribution rate of land use transition carbon sequestration

The interconversion of the two types of land causes changes in the amount of carbon sequestered by the ecosystem. If the land with higher carbon sequestration capacity is transferred out more than the area transferred in, it will lead to a decrease in ecological carbon sequestration capacity, and vice versa. The analysis of the contribution rate of carbon sequestration change by land use conversion is useful to explore the dominant factors causing the change in regional carbon sequestration ecosystem services. The specific calculation method is as follows.

5 $$If, \;\; {S}_{A-B}-{S}_{B-A}>0\left\{\begin{array}{c}If, \;\;{C}_{A}>{C}_{B}, \;\;then\;\;LC=\left({S}_{A-B}-{S}_{B-A}\right)\times \left({C}_{B}-{C}_{A}\right), \;\;LC<0\\ If, \;\; {C}_{B}>{C}_{A}, \;\; then \;\; LC=\left({S}_{A-B}-{S}_{B-A}\right)\times \left({C}_{B}-{C}_{A}\right), \;\;LC>0\end{array}\right.$$

6 $$If, \;\; {S}_{A-B}-{S}_{B-A}<0\left\{\begin{array}{c}If, \;\; {C}_{A}>{C}_{B}, \;\; then \;\; LC=\left({S}_{B-A}-{S}_{A-B}\right)\times \left({C}_{A}-{C}_{B}\right), \;\; LC>0\\ If, \;\; {C}_{B}>{C}_{A}, \;\; then \;\; LC=\left({S}_{B-A}-{S}_{A-B}\right)\times \left({C}_{A}-{C}_{B}\right), \;\; LC<0\end{array}\right.$$

7 $$LCR=\frac{LC}{\sum_{i=1}^{n}LC}$$

where $LC$ represents the change in ecosystem carbon sequestration after interconversion of 2 land classes $A$ and $B$. ${S}_{A-B}$ and ${S}_{B-A}$ represent the area of land class $A$ converted to $B$ and the area of land class $B$ converted to land class $A$. ${C}_{A}$ and ${C}_{B}$ represent the carbon sequestration of land class $A$ and land class $B$ , respectively. $LCR$ represents the contribution of carbon sequestration change from land use conversion, and since carbon sequestration may increase or decrease, it is important to classify positive and negative values of $LC$.

#### Hotspot analysis of carbon supply and demand

To better explore the ecological payment in the ecological deficit area and the fair and sustainable development strategy of the regional ecological environment, the selection of the appropriate ecological compensation area is an important part. Based on previous studies^25^, this study selects hot spot analysis in local spatial autocorrelation statistics to calculate the $\mathrm{Getis Ord Gi}*$ of $CSDR$ to determine the clustering of high $CSDR$ values in space. Hotspots are ecological compensation areas and key areas for receiving ecological payments. The calculation formula of hot spot analysis is as follows.

8 $${G}_{i}^{*}=\frac{\sum_{j=1}^{n}{W}_{ij}{X}_{j}-\overline{X }\sum_{j=1}^{n}{W}_{ij}}{S\sqrt{\frac{n\sum_{j=1}^{n}{W}_{ij}^{2}-{\left(\sum_{j=1}^{n}{W}_{ij}\right)}^{2}}{n-1}}}$$

9 $$S=\sqrt{\frac{\sum_{j=1}^{n}{X}_{j}^{2}}{n}-{\left(\overline{X }\right)}^{2}}$$

where ${G}_{i}^{*}$ is the $\mathrm{Getis Ord Gi}*$ score of the ith region. ${X}_{j}$ is the attribute value of region $j$. $n$ is the total number of counties in the region. $S$ is the standard deviation of all data. ${W}_{ij}$ is the spatial weight between regions $i$ and $j$. $\overline{X }$ is the average of all data. If ${G}_{i}^{*}>0$ and passes the significance test, the higher ${G}_{i}^{*}$ indicates the denser hotspot clustering and the higher the necessity of implementing ecological compensation.

#### Spatial heterogeneity analysis of $CSDR$

Global autocorrelation analysis reveals the spatial correlation of $CSDR$^6^. The value of $Mora{n}^{{\prime}}s \;\;I$ ranges from − 1 to 1. Positive value represents positive spatial correlation, and the larger the value, the more significant the positive correlation and the stronger the spatial agglomeration. Negative values do the opposite. Zero represents the spatially dependent and random distribution of spatial units. The local indicators of spatial association (LISA) explains the spatial heterogeneity or attribute values of geographical phenomena and estimates the spatial extent and location of grouped areas^61^. Through calculation, the clustering results can be divided into low value cluster (Low-Low), high value cluster (High-High), low-value cluster mainly surrounded by high value (Low–High) and high-value cluster mainly surrounded by low value (High-Low). The calculation formula is as follows:

10 $$Mora{n}^{{\prime}}s \; I=\frac{n\sum_{i=1}^{n}\sum_{j\ne 1}^{n}{w}_{ij}({x}_{i}-\overline{x })({x}_{j}-\overline{x })}{(\sum_{i=1}^{n}\sum_{j=1}^{n}{w}_{ij})\sum_{i=1}^{n}{({x}_{i}-\overline{x })}^{2}}$$

11 $${Mora{n}^{{\prime}}s} \; I_{i}=\frac{n({x}_{i}-\overline{x })\sum_{j}{w}_{ij}({x}_{j}-\overline{x })}{\sum_{i}{({x}_{i}-\overline{x })}^{2}}$$

where $n$ is the number of space units. ${x}_{i}$ and ${x}_{j}$ are the $CSDR$ of space units $i$ and $j$, respectively. $\overline{x }$ is the average of the $CSDR$. ${w}_{ij}$ is the spatial matrix.

#### Comparative ecological radiation force and ecological compensation

The ecosystem function basically follows the law of distance decay^62^. In a certain area, the influence of ecosystem services in different areas on external areas within different distances occurs through spatial circulation. The concept of the comparative ecological radiation force (CERF) is introduced into the gravity model in physics, and the interaction relationship of ecosystem services between the two regions is quantitatively studied with reference to the breaking point formula^63^. The breaking point formula for ecosystem service flow is as follows.

12 $$BFES=\frac{{D}_{ds}}{1+\sqrt{{{N}_{d}}/{{N}_{s}}}}$$

where $BFES$ is the ecological radiation force of supply area $s$ to demand area $d$. ${D}_{ds}$ is the distance between demand area $d$ and supply area $s$. ${N}_{s}$ and ${N}_{d}$ are the carbon sequestration of ecosystem services in supply area $s$ and demand area $d$.

To effectively address the problem that the mobility of ecosystem services decreases with increasing distance, an exponential distance decay function is introduced, and a fixed distance weighting function is used to correct ${D}_{ds}$ in Eq. (10) to calculate the flow of carbon sequestration ecosystem services in different supply areas to different values according to the relative size of the demand area. The correction formula is as follows.

13 $${W}_{ds}={e}^{-{D}_{ds}/{H}_{Max}}$$

where ${W}_{ds}$ is the exponential decay distance between the demand area $d$ and the supply area $s$, and ${H}_{max}$ is the maximum distance between each demand area and the supply area.

Finally, the calculation formula of $CERF$ in Xinjiang County is obtained.

14 $${E}_{c}=\frac{{e}^{-{D}_{ds}/{H}_{Max}}}{1+\sqrt{{{N}_{d}}/{{N}_{s}}}}$$

where ${E}_{c}$ is the $CERF$ between regions.

The amount of ecological compensation received by a county is then calculated .

15 $${AEC}_{1}=\frac{{EC}_{1}}{{Total\_EC}_{1}}\times {CSDR}_{1}*P$$

16 $$Tota\_AEC={AEC}_{1}+{AEC}_{2}+\dots +{AEC}_{n}$$

where ${AEC}_{1}$ represents the compensation amount provided from the first demand area. ${EC}_{1}$ indicates the $CERF$ of the first ecological supply area to the first ecological demand area, and ${Total\_EC}_{1}$ indicates the sum of the $CERF$ of all ecological supply areas to the first ecological demand area. ${CSDR}_{1}$ indicates the ecological deficit of the first ecological demand area. $P$ represents the average price of carbon trading. $Tota\_AEC$ represents the total amount of ecological payments received by a supply area from all demand areas.

## Results

### Land use change

#### Space–time pattern of land use

**Figure 4:**
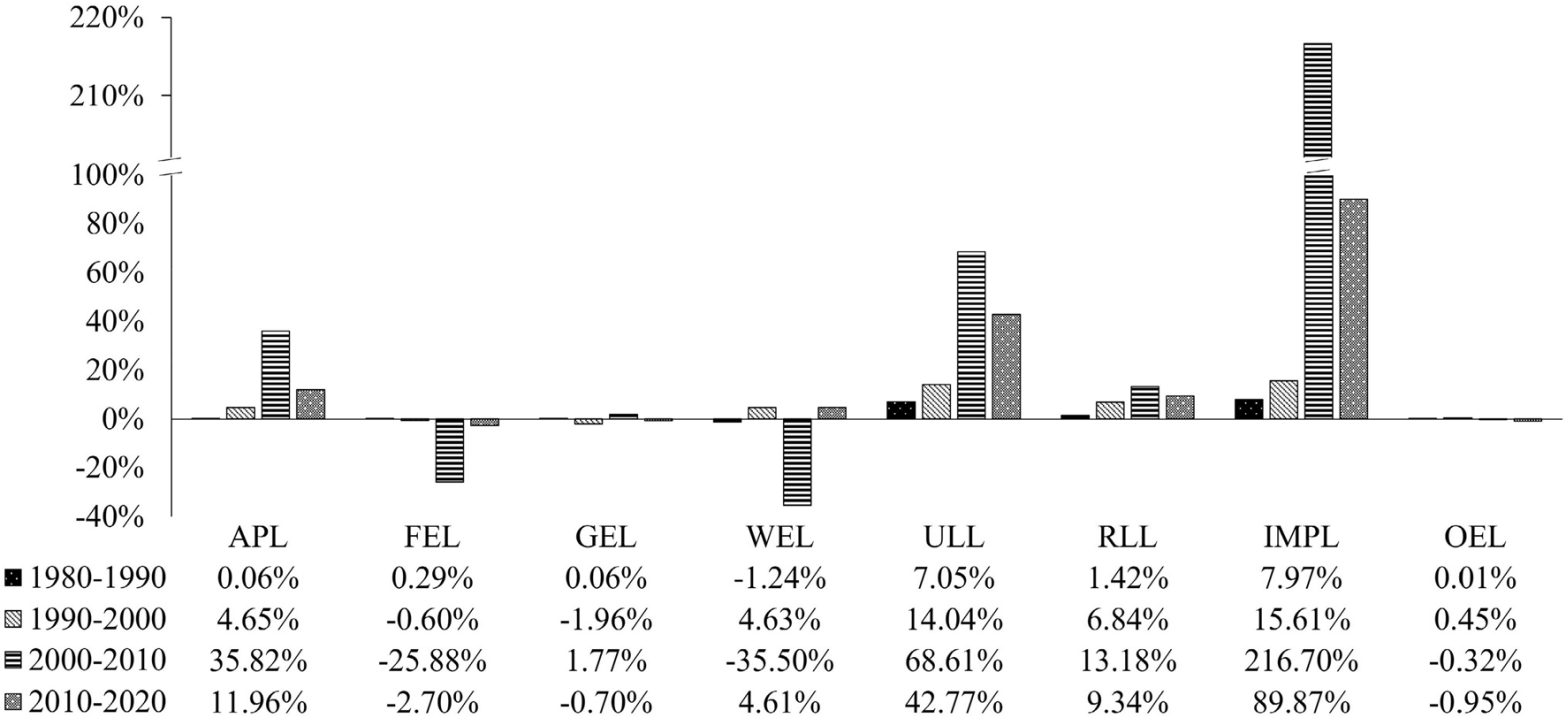
The change range of land use area in Xinjiang (APL: Agricultural production land, FEL: Forestry ecological land, GEL: Grassland ecological land, WEL: Water ecological land, ULL: Urban living land, RLL: Rural living land, IMPL: Industrial and mining production land, OEL: Other ecological land). Software: Excel 2021. URL: https://www.microsoft.com/zh-cn/.

According to Fig. 4 and Supplementary materials 1, in the past 40 years, the living land and production land in Xinjiang have expanded by 2549.76 km^2^ and 35,744.88 km^2^, respectively, with increases of 72.27% and 62.77%. Ecological land occupies 90% of Xinjiang's area, which is dominated by desert and other unused land, followed by grassland. The area of ecological land has shrunk by 38,307.07 km^2^ in the last 40 years, and the desert and oasis margins contain large variations, such as the edges of the Gurbantunggut Desert, the Taklamakan Desert and grassland, and the intersections of these places with oases. The largest change has occurred in industrial and mining production land, followed by urban living land, with increases of 650.61% and 193.84% respectively, compared with 1980; for both land types, the large inflection point began to expand significantly in 2000. The area of agricultural production land has had the largest change, with a direct expansion of 33,526.69 km^2^ in the past 40 years. The spatial distribution of rural living land has some similarity with that of agricultural production land, and the area of rural living land has expanded by 915.63 km^2^. Among the ecological land types, the largest reduction rate and area has been in water ecological land. The overall change in other ecological land has been relatively small, but due to its large area, the area of change has been relatively large. It gradually increased in the first 20 years, then gradually decreased in the next 20 years, and generally decreased in the past 40 years.

#### Land use transition characteristics

In 2000, Xinjiang's land use had obviously different transition characteristics. According to Table 3, in the period 1980–2000, 8687.48 km^2^, 1256.56 km^2^, 699.20 km^2^, and 649.22 km^2^ of grass ecological land, other ecological land, forestry ecological land, and rural living land were transferred to agricultural production land. It is worth noting that 5383.78 km^2^ of agricultural production land was converted to grassland during this period, but the amount of encroachment was clearly not offset compared to the amount of grassland occupied. Degradation from grass ecological land to other ecological land was the largest type of transfer area during this period. Water and forestry ecological land were also being degraded to other ecological land in large quantities. The conversion of other ecological land to these three types of ecological land accounted for 71.36% of the conversion of these three types of ecological land to other ecological land. The expansion of urban and rural living land mainly encroached on agricultural production land and grassland ecological land, and the area of rural living land encroaching on agricultural production land was larger than the area converted into agricultural production land. Land use at this stage can be characterized as follows: in the early stage of the Reform and Opening-up, due to population growth and economic recovery, the agricultural production-led development model caused serious ecological damage.

**Table 3:** Land use transition matrix in Xinjiang from 1980 to 2000 (km^2^).

	APL	FEL	GEL	WEL	ULL	RLL	IMPL	OEL
APL	47,760.66	722.08	5383.78	308.75	146.34	796.40	9.82	1476.67
FEL	699.20	34,611.27	2248.53	195.58	11.02	11.20	0.00	398.00
GEL	8687.48	2009.27	460,142.99	2139.08	70.62	162.59	27.30	12,282.37
WEL	132.58	162.73	1241.61	45,894.46	0.18	3.09	18.67	2648.20
ULL	85.25	1.55	13.50	0.03	729.24	10.04	1.42	1.98
RLL	649.22	4.51	95.36	6.38	17.75	1892.35	0.00	19.36
IMPL	3.88	0.00	1.29	0.01	4.59	0.00	326.82	4.36
OEL	1256.56	545.38	7162.16	3230.30	49.35	33.88	41.55	983,386.86

According to Table 4, during the period from 2000 to 2020, in the mutual transformation between grassland ecological land and other ecological land, the net increase in grassland ecological land was 14,297.80 km^2^. The area of other ecological land converted to grassland, forestry, and water ecological land was obviously larger than the area of ​grassland, forestry, and water ecological land that degraded to other ecological land. These results are diametrically opposed to the situation in the previous stage. The expansion area of agricultural production land has been larger than the area converted into other land. Some rural settlements have been converted into agricultural production land, but this conversion is 366.27 km^2^ less than the conversion of agricultural production land into rural living land, and rural construction land is also expanding on a large scale. In the internal transformation of water, grass and forest ecological land, the degradation of forestland to grassland is significant, and the conversion of forestland to water ecological land and the conversion of water ecological land to grassland are significant. The new urban living land is mainly transformed from agricultural production land, grass ecological land, rural living land and other ecological land. The new rural living land and industrial and mining production land are mainly transformed from agricultural production land, grass ecological land and other ecological land. Based on the above results, at this stage, ecological restoration in most areas of Xinjiang has achieved great results. From the analysis of land conversion, it can be seen that land use has gradually shifted from competition and conflict to coordination, optimization, and reorganization.

**Table 4:** Land use transition matrix in Xinjiang from 2000 to 2020 (km^2^).

	APL	FEL	GEL	WEL	ULL	RLL	IMPL	OEL
APL	50,558.58	1246.78	3857.21	353.73	739.45	1736.67	121.53	658.48
FEL	3232.66	14,033.54	17,615.33	483.15	74.97	90.24	13.32	2507.30
GEL	23,569.87	10,105.15	326,908.63	4263.82	412.04	421.82	576.90	109,972.64
WEL	660.55	333.42	8380.98	22,945.48	21.06	11.76	69.30	19,313.43
ULL	63.58	4.09	15.49	2.66	873.90	45.89	13.83	9.65
RLL	1370.40	89.01	193.94	15.00	112.04	1072.91	20.54	35.72
IMPL	24.24	1.09	35.89	4.86	20.24	12.66	133.11	193.48
OEL	10,650.49	1627.95	124,270.44	6855.09	223.42	208.59	1610.60	854,661.74

#### Land use change tracking analysis

**Figure 5:**
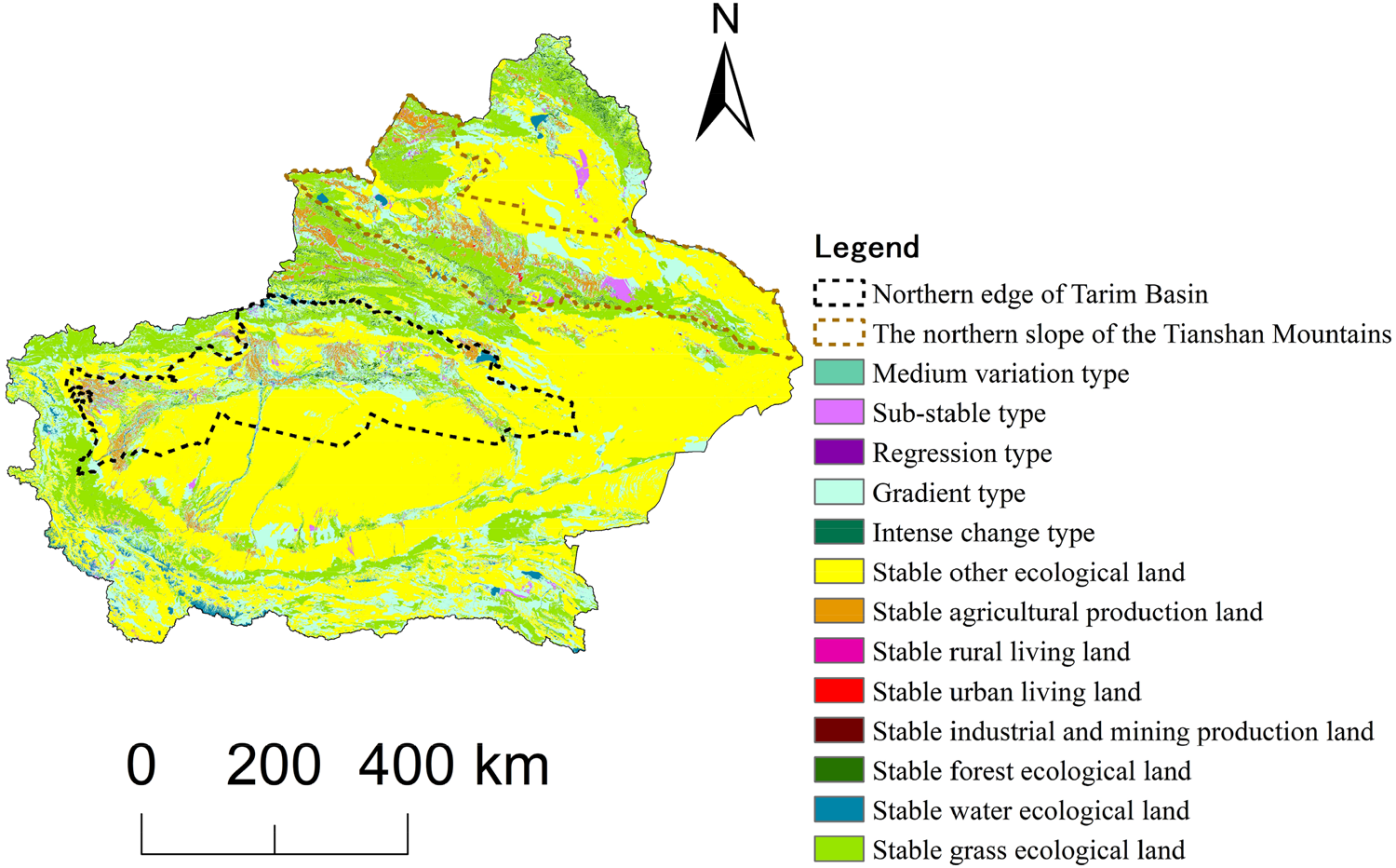
Types of land use change trajectories in Xinjiang from 1980 to 2020. Map generated with ArcGIS 10.8 (ESRI). URL: https://support.esri.com/en/Products/Desktop/arcgis-desktop/arcmap.

From 1980 to 2020, there were 3606 land use change trajectories in Xinjiang (Fig. 5). The land use change trajectory of each raster image element was identified. The area of the stable land use change trajectory accounted for 75.55% of the total area of Xinjiang, which was the largest type (Table 5). The area of the gradient land use change trajectory accounted for 22.01%, which was the most dominant change trajectory in Xinjiang over the past 40 years. Except for the interior of the Taklimakan Desert, the interior of the Gurbantunggut Desert, the interior of the Gobi in eastern Xinjiang, and some mountainous areas, the gradient type trajectory is widely distributed throughout the country. On the whole, the northern slope of the Tianshan Mountains and the northern margin of the Tarim Basin have the most types of changes and the largest areas of change. These two areas in Xinjiang also have relatively concentrated distributions of productivity and population. On the northern edge of the Tarim Basin, the net transfer of ecological land to production land is 14,730.40 km^2^, which is 4024.07 km^2^ more than the area on the northern slope of the Tianshan Mountains. In the internal transformation of ecological land, 13,556.53 km^2^ of grassland, forest and water ecological land in the northern edge of the Tarim Basin has been transferred to other ecological land. The northern slope of the Tianshan Mountains presents the opposite pattern.

**Table 5:** Pixel-by-pixel tracking analysis and statistics of five phases of land use change in Xinjiang.

Type	Percentage of area/%	Main distribution area
Stable type	75.55	Distributed everywhere
Sub-stable type	1.55	The northern slope of the Tianshan Mountains, around the Tarim Basin, along the Tarim River, and around the Junggar Basin
Regression type	0.02	The northern slope of the Tianshan Mountains, the northern and western margins of the Tarim Basin
Gradient type	22.01	Except for stable deserts, Gobi, and mountains, it is distributed in other places
Medium variation type	0.86	The northern slope of the Tianshan Mountains, around the Tarim Basin, and around the Junggar Basin
Intense change type	0.01	The northern slope of the Tianshan Mountains, the northern margin of the Tarim Basin, and the northern margin of the Junggar Basin

### Changes in carbon supply and demand

#### Changes in carbon sequestration and its relationship to land use

**Figure 6:**
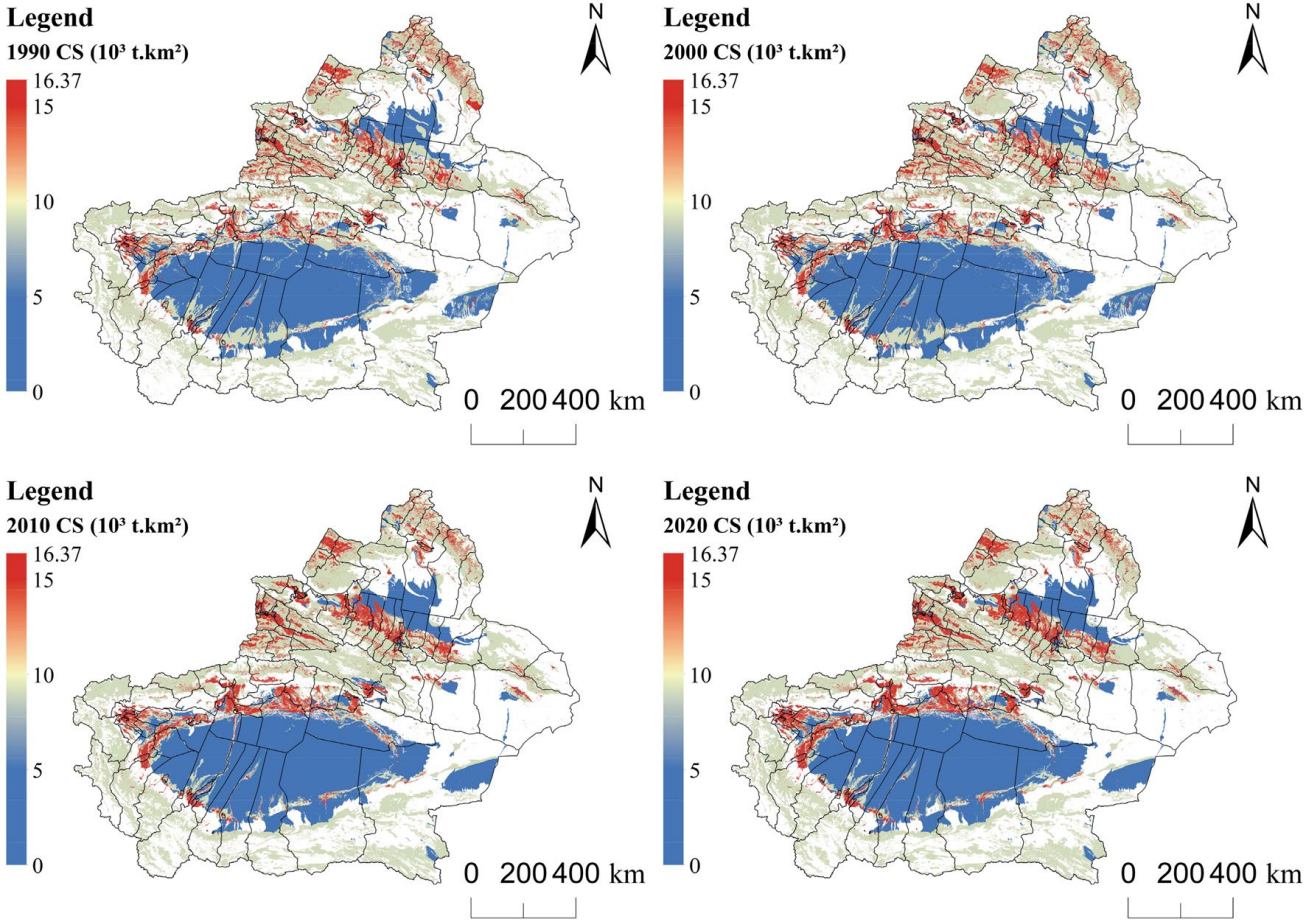
Changes in carbon sequestration in Xinjiang ecosystems. Map generated with ArcGIS 10.8 (ESRI). URL: https://support.esri.com/en/Products/Desktop/arcgis-desktop/arcmap.

**Figure 7:**
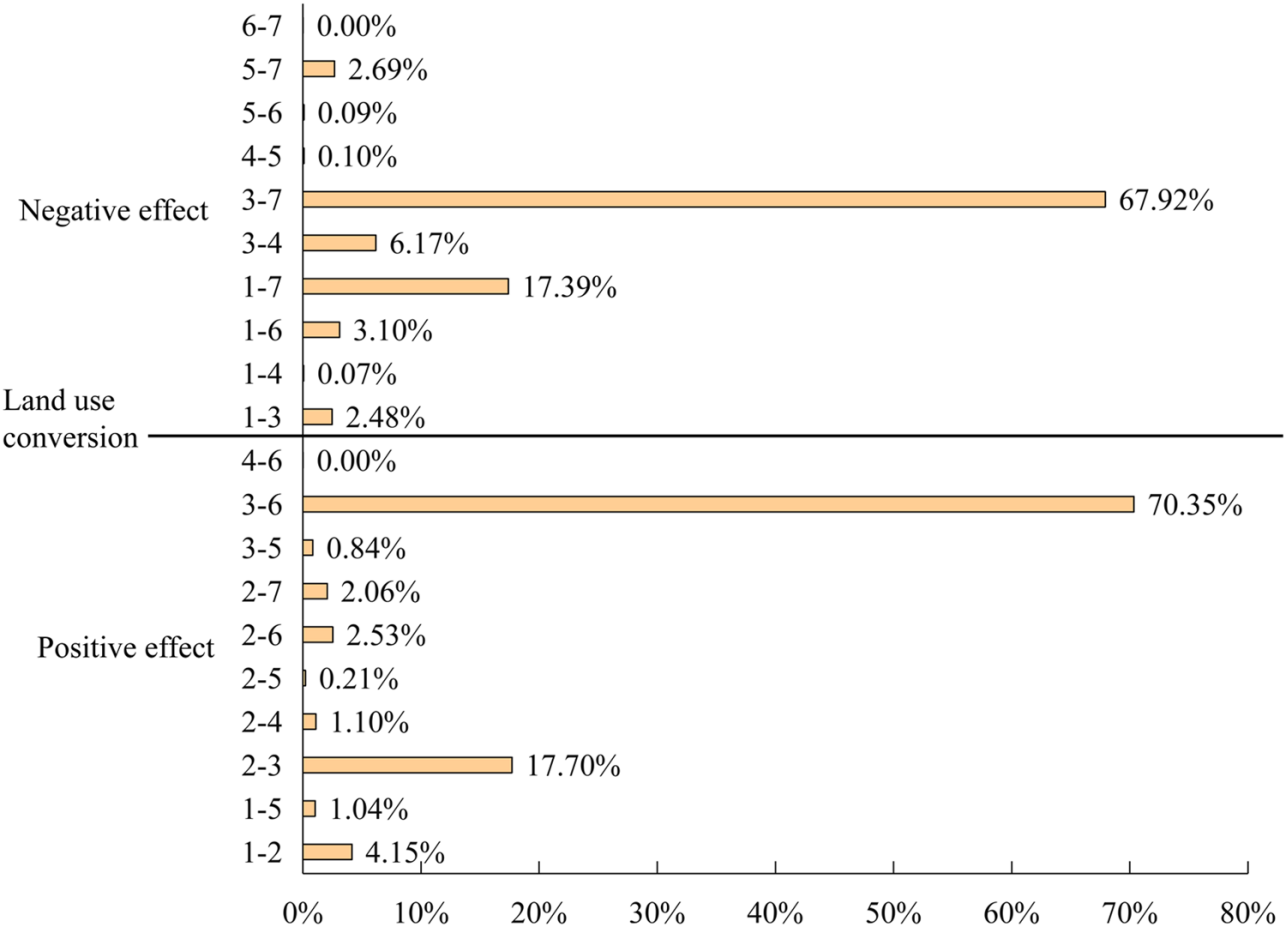
Effects of land use change on carbon sequestration in ecosystems (1: Agricultural production land. 2: Forestry ecological land. 3: Grassland ecological land. 4: Water ecological land. 5: Artificial surface. 6: Desert. 7: Other ecological land). Software: Excel 2021. URL: https://www.microsoft.com/zh-cn/.

**Figure 8:**
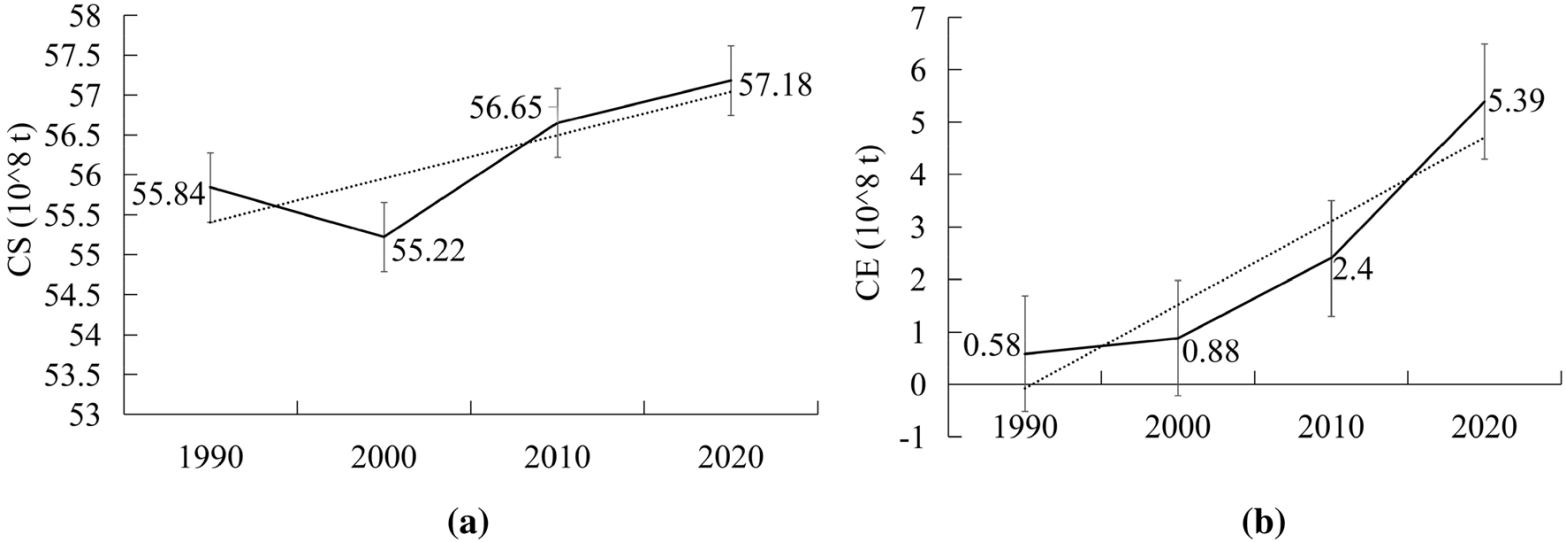
Changes of carbon sequestration (**a**) and carbon emission (**b**) in Xinjiang. Software: Excel 2021. URL: https://www.microsoft.com/zh-cn/.

To scientifically evaluate the carbon sequestration capacity of Xinjiang's ecosystem, the desert is separated from other ecological land for research. According to Figs. 6, 8a, and Supplementary materials 2, the carbon sequestration in Xinjiang first decreased and then increased during the past 30 years. Ruoqiang county in the lower right corner of Xinjiang had the largest carbon sequestration in Xinjiang over time (Fig. 10b). Ruoqiang is the largest county in Xinjiang, with a low population density and extensive distribution of ecological land such as deserts, grasslands, and woodlands. Tianshan county had the smallest carbon sequestration over the time; this county has a small area and is an urban area developed earlier in Xinjiang with many man-made buildings. The distribution of changes in carbon sequestration in Xinjiang from 1990 to 2020 paralleled the changes in land use, and the carbon sequestration in areas with frequent human activities has been significantly reduced. Forestland has the largest carbon sequestration in Xinjiang, followed by grassland and agricultural production land. There is a direct relationship between land use change and carbon sequestration change (Fig. 7, Supplementary materials 3). The interconversion between 10 pairs of land types, led by the conversion of other unused land to grassland and other unused land to cropland, resulted in increased carbon sequestration. The interconversion between 10 pairs of land types, led by the conversion of grassland to desert and woodland to grassland, resulted in lower carbon sequestration.

#### Changes in carbon emissions

**Figure 9:**
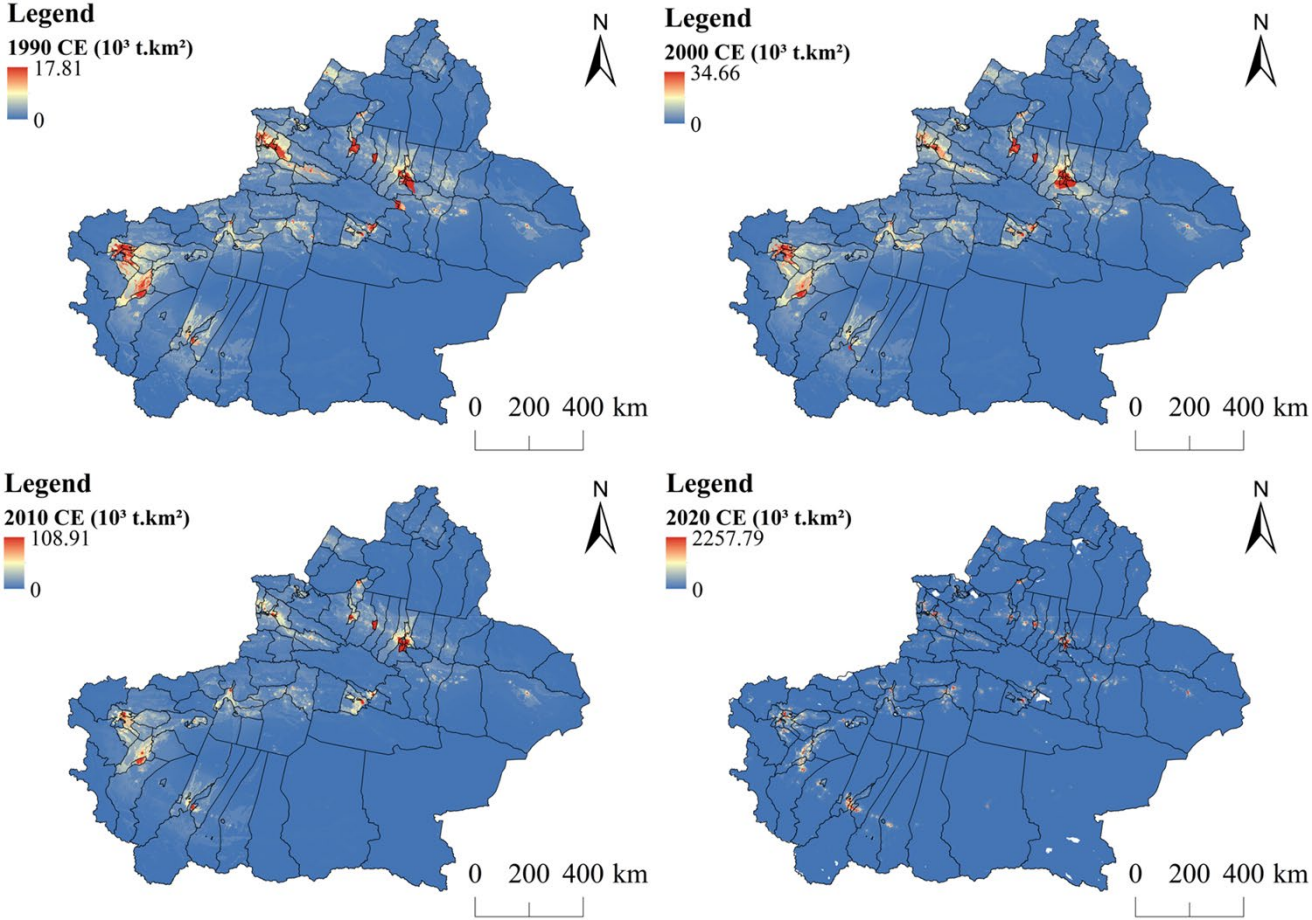
Changes in Xinjiang Carbon Emissions. Map generated with ArcGIS 10.8 (ESRI). URL: https://support.esri.com/en/Products/Desktop/arcgis-desktop/arcmap.

**Figure 10:**
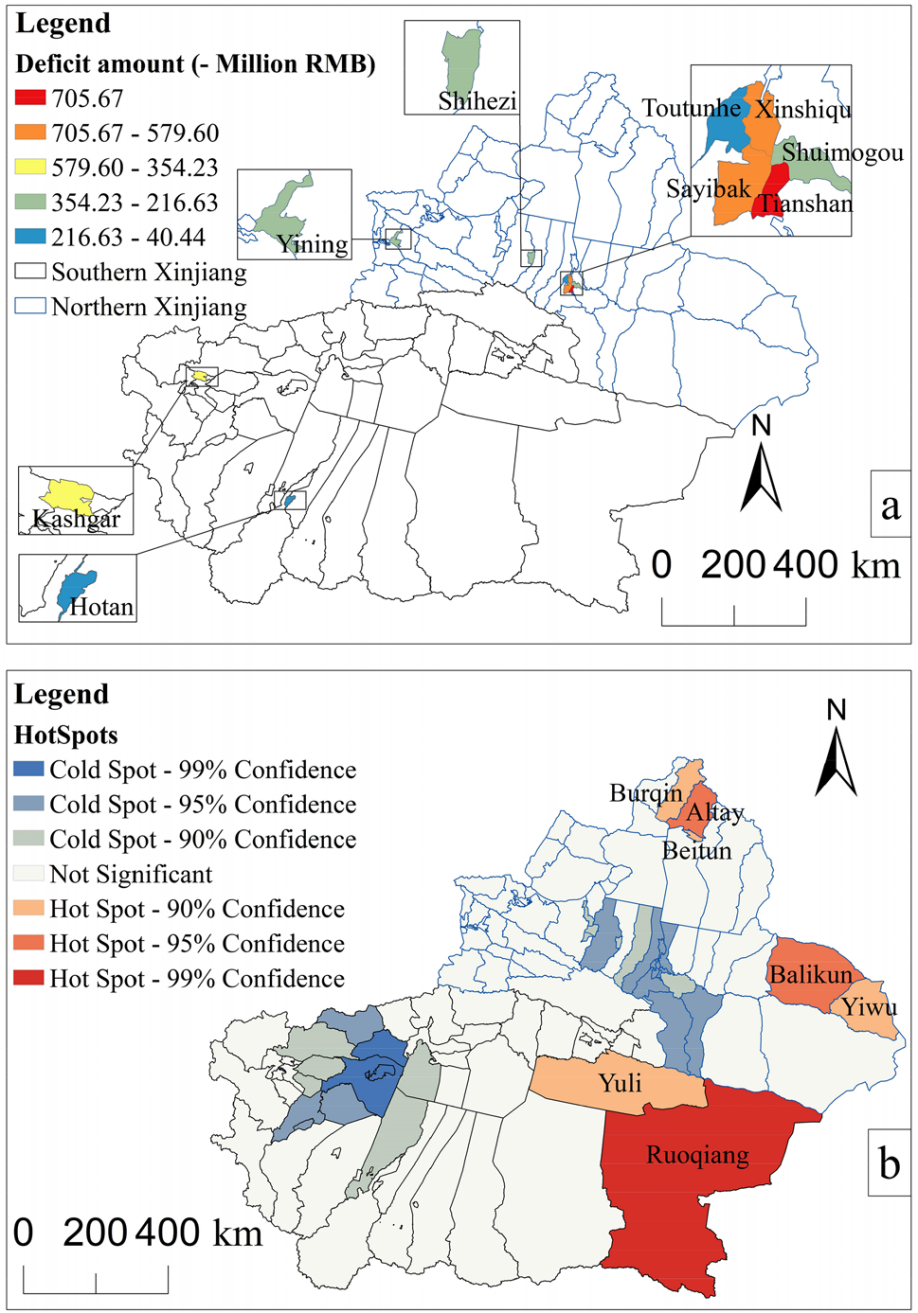
Carbon sequestration ecological deficit areas (**a**) and hotspots (**b**). Map generated with ArcGIS 10.8 (ESRI). URL: https://support.esri.com/en/Products/Desktop/arcgis-desktop/arcmap.

The per capita carbon emissions in Xinjiang surged from 3.93 t a^−1^ in 1990 to 220.58 t a^−1^ in 2020, leading to an overall intense growth in Xinjiang's overall carbon emissions, with a direct increase of 481.37*10^6^ t in 30 years. According to Figs. 8b, 9, and Supplementary materials 2, the areas with large carbon emissions over the years are concentrated in the northern slope of the Tianshan Mountains, the northern and western margins of the Tarim Basin, and the urban areas of Altay (Fig. 10b). The areas with lower carbon emissions are the two major desert regions and the sparsely populated areas of eastern Xinjiang. The county with the largest increase in carbon emissions is Beitun, which is below Altay in the northwest corner. Beitun is home to the Xinjiang Production and Construction Corps. It was sparsely populated before development. With the strengthening of production and construction, it has a strong impact on carbon emissions. In addition, Tianshan, Sayibak, and Xinshiqu of Urumqi have large increases in carbon emissions.

### Ecological compensation scheme

#### Ecological demand area and supply area

Based on the carbon supply and demand in 2020, the nine ecological deficit districts and counties in 2020 were selected as demand areas (Fig. 10a). In 2020, most areas in Xinjiang had ecological surpluses. Ruoqiang, Qiem and Hejing in southeastern Xinjiang had large carbon surpluses. Due to the large area of Xinjiang and the large environmental differences between southern and northern Xinjiang, certain differences in land use transitions and economic development can be found between northern and southern Xinjiang. This study divides Xinjiang into southern and northern Xinjiang for ecological compensation research. Through hot spot analysis of the ecological supply and demand of carbon sinks in southern and northern Xinjiang, the hot spots in Fig. 10b were obtained. To fully consider the ecological background of the hotspot area and whether the ecological surplus can meet the overall demand of the demand area, six ecological supply areas were selected.

#### CERF and compensation schemes

According to the calculation model, the CERF (Table 6) of carbon sink ecosystem services to demand areas through spatial circulation is obtained. In the southern Xinjiang region, Ruoqiang and Yuli have a larger CERF than Hotan, and Yuli has the largest CERF overall. In northern Xinjiang, the CERF of the four carbon sequestration sources to Tianshan is relatively large, and the CERF to Yining is relatively small. At the same time, Altay and Burqin have a relatively large CERF to Xinshiqu. The overall CERF of Balikun is the largest, and the overall CERF of Yiwu is the smallest. The area of Tianshan District is small, with less natural carbon sequestration of ecological land and a high demand for ecosystem services. Coupled with its moderate location for supply, the comparative ecological radiation of the carbon sequestration source sites to Tianshan is high. However, Yining is far from carbon sequestration sources, and its ecological environment quality is relatively good due to the influence of humid Atlantic oceanic air currents, and its demand for ecosystem services is relatively low. The areas with large CERF have the largest total overflow of carbon sink ecosystem services and the highest ecological contribution to the surrounding areas. The overflowing carbon sink ecosystem services gradually decrease with the increasing of distance. Balikun and Yuli County are the key compensation areas in northern and southern Xinjiang respectively.

**Table 6:** CERF in Xinjiang carbon sequestration ecological supply and demand area.

	Supply area	Ruoqiang	Yuli			Total
Southern Xinjiang (demand area)	Kashgar	0.33	0.37	**–**	**–**	0.70
	Hotan	0.44	0.45	**–**	**–**	0.89
	Total	0.77	0.82	**–**	**–**	**–**
Northern Xinjiang (demand area)	Supply area	Balikun	Yiwu	Altay	Burqin	Total
	Tianshan	0.62	0.50	0.58	0.55	2.26
	Sayibak	0.59	0.45	0.54	0.52	2.10
	Xinshiqu	0.61	0.48	0.58	0.55	2.21
	Shuimogou	0.61	0.48	0.56	0.53	2.19
	Toutunhe	0.60	0.47	0.57	0.54	2.18
	Yining	0.36	0.27	0.43	0.43	1.50
	Shihezi	0.53	0.40	0.56	0.54	2.02
	Total	3.91	3.06	3.82	3.66	**–**

**Figure 11:**
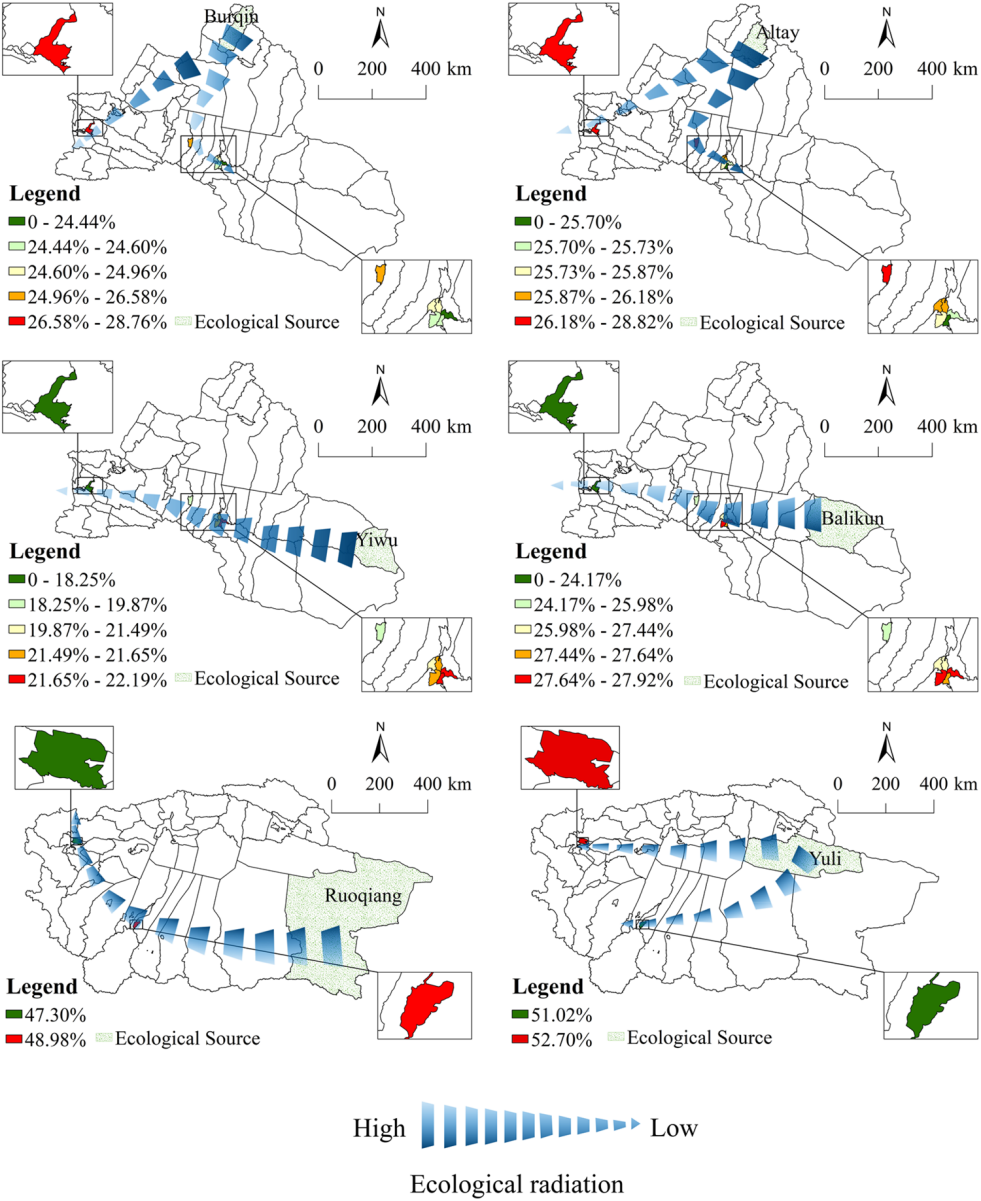
The proportion of ecosystem services provided by different ecological sources to the total amount received by the county. Map generated with ArcGIS 10.8 (ESRI). URL: https://support.esri.com/en/Products/Desktop/arcgis-desktop/arcmap.

According to Fig. 11, among the carbon sink ecosystem services obtained in the five ecological deficit areas in Urumqi, the carbon sink ecosystem services provided by Balikun through spatial circulation are the highest, exceeding 27%. Among the carbon sink ecosystem services obtained by Yining and Shihezi, Altay provided the most, exceeding 27%. Because Yiwu is far from the demand area and its carbon sequestration capacity is not as good as that of Balikun, Yiwu provided the least ecological services among all demand areas in northern Xinjiang. In the southern Xinjiang region, among the carbon sink ecosystem services obtained by Kashgar and Hotan, Yuli is the largest provider, accounting for more than 50% of the carbon sink ecosystem services it obtained. However, Ruoqiang County's flow of carbon sink ecological services to the two ecological deficit counties should not be underestimated, as its lowest contribution is only 5% lower than that of Yuli. According to Table 7, in the northern Xinjiang region, Tianshan paid the highest total amount, and Balikun received the most compensation. In the southern Xinjiang region, Kashgar paid the highest total amount, and Yuli received the most compensation. Different areas received different amounts of money, as the payment amount of demand areas to different supply areas is calculated according to the proportion of different ecosystem services received.

**Table 7:** Ecological payment and ecological compensation program (10^8^ yuan).

	Balikun	Yiwu	Altay	Burqin	Ruoqiang	Yuli	Total
Tianshan	1.95	1.57	1.81	1.73	–	–	− 7.06
Sayibak	1.63	1.27	1.52	1.44	–	–	− 5.86
Xinshiqu	1.59	1.25	1.51	1.44	–	–	− 5.80
Shuimogou	0.76	0.60	0.70	0.67	–	–	− 2.73
Toutunhe	0.11	0.09	0.11	0.10	–	–	− 0.40
Yining	0.52	0.40	0.62	0.62	–	–	− 2.17
Shihezi	0.69	0.53	0.73	0.71	–	–	− 2.66
Kashgar	–	–	–	–	1.68	1.87	− 3.54
Hotan	–	–	–	–	0.61	0.64	− 1.25
Total	7.26	5.70	7.01	6.71	2.29	2.51	–

## Discussion

### Reasons for changes in carbon sinks and carbon sinks carbon emissions

LUCC plays an important role in terrestrial carbon sequestration ecosystem services, and carbon stocks will increase when land types with low biomass content are converted to land types with higher biomass^64^. Thus, land use transitions profoundly affect changes in carbon sequestration ecosystem services, and there are similarities in their drivers. Taken together, there are multiple influencing factors. Partly due to changes in natural elements such as climate, Xinjiang rapidly warmed by 0.30 °C/decade between 1961 and 2018 (p < 0.01), and annual precipitation showed a gradual incremental trend. The tendency of the climate to become warmer and wetter has directly led to changes in the natural environment of Xinjiang, resulting in glacial retreat, reduced snowfall, increased river runoff, lake expansion, and changes in vegetation cover^65^. Another part of the reason is that since the Reform and Opening-up in 1978, Xinjiang's GDP has been increasing rapidly and the population has been increasing^66^, so the land directly related to human activities, such as agricultural production land, urban living land, rural living land, industrial and mining production land, has been expanding. Since the Reform and Opening-up in 1978, the dominant policy stages in Xinjiang can be divided into four phases: land reclamation (1978–1990), agricultural resource development (1990–2000), western development (2000–2010), and counterpart assistance to Xinjiang (2010-present), and the national policy orientation at different stages has had a huge impact on land use changes^67^. From 1990 to 2000, this period was the period of economic development and population growth in Xinjiang. During this period, Xinjiang's food security problems were prominent and agriculture developed rapidly. At this stage, the lack of ways to achieve national land reclamation led to massive deforestation and the improvement of other ecological lands, thus leading to a rapid reduction in the amount of carbon sequestered by the ecosystem. In 1998, China established the Land Consolidation Center of the Ministry of Land and Resources, and then the country gradually entered a period of arable land quantity protection and comprehensive quality construction. A series of projects such as land development and consolidation, reclamation, balancing arable land occupation and compensation, and linking the increase and decrease of construction land, have been put into practice^68^. This process also provides a basis for explaining the conversion of land use types such as rural subsistence land to productive agricultural land and the large expansion of productive agricultural land in the results of this paper. In 2013, the Chinese government “introduced ecological civilization policy”, and Xinjiang entered a new stage in terms of reforestation and ecological restoration. Sustainable development is incorporated into Xinjiang's development goals. Xinjiang is an underdeveloped region in China, but its energy resources are relatively abundant. Through our research, we found that the degradation of grassland and cropland in Xinjiang is the main reason for the decline in carbon sequestration, while the change form desert to grassland and form grassland to woodland is the main reason for the increase in carbon sequestration, demonstrating the importance of woodland, agricultural production land and grassland for ecological sustainability and ecological protection in Xinjiang.

**Figure 12:**
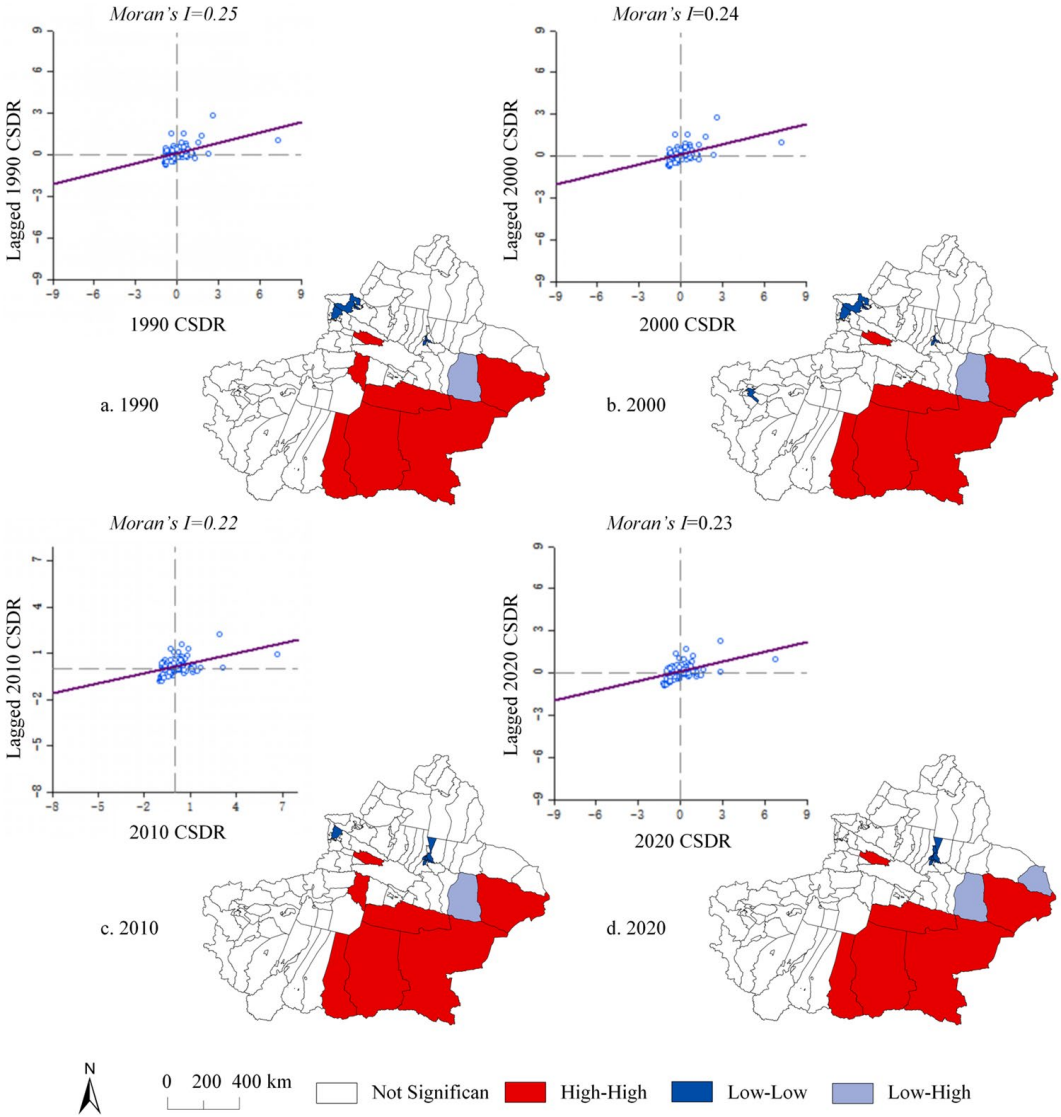
The Moran scatter and LISA cluster graph of CSDR in Xinjiang from 1990 to 2020. Map generated with ArcGIS 10.8 (ESRI). URL: https://support.esri.com/en/Products/Desktop/arcgis-desktop/arcmap.

To promote economic development, the local government chose an unsophisticated development approach in the early stage of development, using energy consumption as the driving force for economic growth, and although the effect of this approach has been significant, carbon emissions are growing rapidly^69^. The carbon emissions in Xinjiang have been increasing rapidly since 2000, with an average annual growth rate of 10.24%^70^. The growth of carbon emissions is similar to the results of this paper, but the difference is that the growth rate and carbon emissions shown in this paper have slightly higher values. This error reflects differences in the carbon emissions accounting process^71^. The CSDR in Xinjiang has a certain spatial clustering phenomenon in 1990–2020, with high value areas tending to be concentrated and low value areas tending to be adjacent to each other (Fig. 12). In the southeastern part of Xinjiang, there is a stable "high-high" clustering, and this area is also the area with high carbon sequestration capacity extracted from this study. Several counties in the central part of northern Xinjiang show stable "low-low" clusters, and these are also the high carbon emission areas extracted from this study, which are the main ecological deficit areas.

### Ecological protection and ecological compensation

The United Nations has adopted "reducing inequalities within and among countries" (SDGs10) and "protecting, restoring and promoting sustainable use of terrestrial ecosystems, sustainably managing forests, combating desertification, halting and reversing land degradation and halting biodiversity loss" (SDGs15) as Sustainable Development Goals (SDGs). One way to achieve the sustainable development of the carbon ecosystem is to improve the carbon sequestration capacity of the ecosystem, and another way is to reduce carbon emissions in the industry (including energy conservation, efficiency enhancement, development, and utilization of new energy and other measures). Of these options, enhancing terrestrial carbon sinks is considered to be one of the most effective ways to mitigate climate change^72^. The Chinese government has implemented a series of ecological protection and restoration projects since the late 1970s, such as the “Three-Northern Protection Forests Project” and “Returning Cultivated Land to Forests”, and recent studies have shown that the implementation of national ecological restoration projects has improved ecosystem services and made a significant contribution to the mitigation of CO_2_ emissions in China^73^. Grasslands in arid regions show obvious carbon sinks characteristics, and grassland carbon sinks mainly occur in natural and low-intensity grassland types^74^. In Xinjiang, human activities should be strictly prohibited from encroaching on nature reserves that maintain biodiversity. The capacity of ecosystem services should be improved through reforestation, reforestation and other ecological projects. At the same time, at the border between oasis and desert, the invasion of sandy areas should be prevented to improve biodiversity. In the oasis arable land, the layout between "water resources, energy and food production" should be planned reasonably. In the case of limited water resources in arid areas, water resources should be effectively converted into ecosystem carbon sink services through agricultural activities or ecological restoration projects. In addition, it is important to focus on the fairness and reasonableness of ecological payments and compensation between different regions. The evaluation framework of ecological compensation mainly includes the subject and object of compensation, the determination of compensation standards and the construction of compensation methods. This is reflected in the establishment of scientific and feasible compensation standards, feasible compensation methods and relevant responsible mechanisms^75^. Carbon trading in Xinjiang is still in its initial stage. With its low population density and large ecological area, Xinjiang is still an important carbon sequestration depot for China and the world, despite the gradual strengthening of its carbon emissions. Ecological compensation for Xinjiang will promote sustainable ecological and economic development in Xinjiang, but the principle of regional and environmental equity should be emphasized when allocating the compensation amount, and ecological compensation should be used to promote better human well-being and ecological conservation.

### Methodological limitations and challenges for future research

As an underdeveloped region in China, Xinjiang lacks high-precision geographic data. Therefore, carbon emissions in this study were calculated according to per capita carbon emissions and population density. However, in practice, the per capita carbon emissions in different regions may vary greatly due to the level of economic development, living standards and industrial structure^52^. In Xinjiang, areas with a concentrated population are also more economically active and have higher carbon emissions. But there are also special places, such as some of the Gobi in Xinjiang, where the population density is low, but the carbon emissions are high, because of the development of oil. In addition, the economic level of counties in northern Xinjiang is generally higher than those in southern Xinjiang, and their per capita carbon emission intensity is also greater. Considering only net carbon emissions as a baseline value for carbon offsets may result in excessive payments in some areas. If researchers have high precision data, they can use GDP, carbon intensity, carbon efficiency and other indicators to form a revised model, setting a carbon emission threshold for each region^61^. At present, ecological payment is mainly dominated by the government, and the factors such as the ability to pay, willingness to pay, conflicts between different stakeholders and regional consumption coefficient are less considered^26^. More specifically, this study was conducted with less consideration of regional differences in ecological compensation standards and with passive compensation in neighboring areas. Future research may consider introducing lower limit, upper limit and regional difference adjustment coefficient of ecological compensation. The study was conducted on administrative boundaries, ignoring the openness of ecosystems and the mobility of ecosystem services in Xinjiang and its surrounding areas. Future studies can integrate the knowledge of geography, meteorology, ecology and economics, and take different scale catchments as research areas to elaborate the supply and demand, flow path, velocity, cost effect and loss of ecosystem services, so as to provide a basis for formulating reasonable ecosystem management policies and ecological compensation schemes. This study is a meaningful attempt to incorporate carbon emissions, carbon sinks and ecosystem service flows into ecological compensation in the context of land use transition. This study explores and improves the relevant theories and has practical implications for sustainable development. However, in the study of ecosystem services and environmental justice, achieving equitable distribution of ecosystem service benefits is still a complex issue.

## Conclusions

The research value of this study is to resolve the differences in the balance of carbon sinks and carbon emissions in Xinjiang counties in the context of land use transition, and to reveal the impact brought by the regions with more inter-regional ecosystem service surpluses to the demand areas. This study attempts to use modified ecological radiative forcing to carve out the ecological radiation of supply and demand areas, as a way to simulate the amount of ecosystem service recharge provided by supply areas to demand areas, and to account for issues such as ecological compensation standards and fund allocation between supply and demand areas. The main conclusions are as follows.

1. This study reveals the characteristics of land use transition in Xinjiang. During the period from 1980 to 2000, the main land use conflicts brought about by population increase and economic recovery existed between agricultural production land and rural living land, grassland, forestland and other ecological land. In the internal transformation of ecological land, the conversion of other ecological land into grassland, woodland and water accounted for 71.36% of the area of these three types of land converted into other ecological land, indicating that ecological land is degrading as a whole. From 2000 to 2020, ecology gradually became protected. At this stage, the net conversion of other ecological land to grassland increased greatly. In addition, the area of other ecological land converted into grass, forestry and water ecological land became significantly larger than the area of these three types of ecological land degraded into other ecological land. Xinjiang's land use has gradually shifted from competition and conflict to coordination, optimization, and reorganization.
2. There is a relationship between land use transitions in Xinjiang and changes in ecosystem carbon sequestration. The reduction of ecological land with high carbon sequestration capacity in the early stage reduced the amount of carbon sequestration. In the later stage, various ecological projects were fully implemented, and changes in natural climate gradually increased the carbon sequestration capacity of the ecosystem. Conservation of cropland and grassland is important to enhance the carbon sequestration capacity of dryland ecosystems. However, with the increase in population and the enhancement of economic and social activities, some regions began to experience ecological deficits in 2010.
3. If an ecological supply area is larger and closer to the demand area, and has a high carbon sequestration capacity, its comparative ecological radiation force to the ecological deficit area is also higher. Based on the comparative ecological radiation force, the proportion of carbon sink ecosystem services obtained by each deficit area from different ecological sources can be obtained, so as to determine the distribution scheme of ecological compensation. In northern Xinjiang, Tianshan was the county that paid the largest amount of ecological compensation, and Balikun was the county that received the largest amount of ecological compensation. In southern Xinjiang, Kashgar was the county that paid the largest amount of ecological compensation, and Yuli is the county that received the largest amount of ecological compensation.

## Supplementary Information

Supplementary Information 1.

Supplementary Information 2.

Supplementary Information 3.

## Data Availability

The datasets used and/or analyzed during the current study are available from the corresponding author on reasonable request.
