# Author Correction: Carbon sinks and carbon emissions balance of land use transition in Xinjiang, China: differences and compensation

**DOI:** 10.1038/s41598-023-28537-9

**Published:** 2023-01-25

**Authors:** Kui Luo, Hongwei Wang, Chen Ma, Changrui Wu, Xudong Zheng, Ling Xie

**Affiliations:** 1 College of Geography and Remote Sensing Sciences, Xinjiang University, Urumqi, 830017 China; 2 Xinjiang Key Laboratory of Oasis Ecology, Xinjiang University, Urumqi, 830017 China; 3 School of Geography and Ocean Science, Nanjing University, Nanjing, 210023 China

Correction to: *Scientific Reports* 10.1038/s41598-022-27095-w, published online 27 December 2022

The original version of this Article contained an error in the legends of Figures 1, 4, 7 and 8, where there provided link was incorrectly given as “https://www.microsof.com/zh-cn/” instead of “https://www.microsoft.com/zh-cn/”.

Additionally, the provided link in the legend of Figure 3 was incorrect. The legend,

“Types of land use change trajectory. Software: PowerPoint 2021. URL: https://support.esri.com/en/Products/Desktop/arcgis-desktop/arcmap”

now reads:

“Types of land use change trajectory. Software: PowerPoint 2021. URL: https://www.microsoft.com/zh-cn/.”

Lastly, this Article contained an error in the legend of Figure 12, where the provided date was incorrect.

“The Moran scatter and LISA cluster graph of CSDR in Xinjiang from 1990 to 2015. Map generated with ArcGIS 10.8 (ESRI). URL: https://support.esri.com/en/Products/Desktop/arcgis-desktop/arcmap.”

now reads:

“The Moran scatter and LISA cluster graph of CSDR in Xinjiang from 1990 to 2020. Map generated with ArcGIS 10.8 (ESRI). URL: https://support.esri.com/en/Products/Desktop/arcgis-desktop/arcmap.”

The original Article has been corrected.

